# The Medical Inspection of Children in Public Elementary Schools

**Published:** 1907-12-07

**Authors:** 


### THE MEDICAL INSPECTION OF CHILDREN IN PUBLIC ELEMENTARY SCHOOLS.

The Memorandum issued by Sir Robert Morant on behalf of the Board of Education under Section 13 of the Education (Administrative Provisions) Act, 1907, is now in the hands of the Local Authorities. It is not too much to say that no document of like importance or of such moment in the development of Public Health Administration has been published for many years.

For some years past evidence has been accumulating that there exists in certain classes of the English people a somewhat high degree of physical unfitness which calls for amelioration, and, as far as possible, for prevention. The Legislature resolved that to grapple effectively with this problem, or at least part of it, it was necessary first to improve the health conditions, both personal and in regard to environment, of the children of the nation.

The Board desire, therefore, at the outset to emphasise that this new legislation aims not merely at a physical or anthropometric survey or at a record of defects disclosed by medical inspection, but at the physical improvement, and, as a natural corollary, the mental and moral improvement of coming generations. The broad requirements of a healthy life are comparatively few and elementary, but they are essential, and should not be regarded as applicable only to the case of the rich. In point of fact, if rightly administered, the new enactment is economical in the best sense of the word. Its justification is not to be measured in terms of money, but in the decrease of sickness and incapacity among children and in the ultimate decrease of inefficiency and poverty in after-life arising from physical disabilities.

It would be incorrect to describe it as revolutionising the local administration of public health, for it is of the essence of the direction given by the Board of Education that in pursuance of the provisions of the Act there shall be no dislocation or supplanting of existing organisation in Public Health Departments, but rather an harmonious expansion in accord with the national needs which it has been shown demand satisfaction in the directions indicated in the Memorandum.

Nothing is more admirable than the wise discrimination and comprehensive outlook with which the local organisation of the work is indicated.

The duty of carrying out the actual inspection has necessarily been entrusted by Parliament to the Local Education Authorities and not to the Board. Each Authority must therefore in due course appoint such medical officers or additional medical assistance as may be required for the purpose. Some time must inevitably elapse before all Authorities have their arrangements in working order, but it should be carefully borne in mind that, although the work is begun gradually, the initial organisation established by each Authority should admit of such expansion as will secure the thorough and efficient administration of the Act. In subsequent paragraphs some general guidance is given as to the minimum amount of inspection required.

In view of the varied influences which affect, directly or indirectly, the health of the children of the nation, it is manifestly of the highest importance that the administration of this Act should rest upon a broad basis of public health, and should not only secure for Local Education Authorities as much freedom as is consistent with adequate uniformity in the presentation of results for comparative purposes, but should also use to the utmost extent the existing machinery of medical and sanitary administration, developing and supplementing it as required, rather than supplanting it by bringing into existence new agencies, partially redundant and possibly competing.

An Integral Factor in the Health of the Nation.

The Board view the entire subject of school hygiene not as a speciality or as a group of specialities existing by and of themselves, but as an integral factor in the health of the nation. The application of this principle requires that the work of medical inspection should be carried out in intimate conjunction with the Public Health Authorities and under the direct supervision of the medical officer of health. Provided that the principle of co-ordination of the medical services is secured in practice, and that the requisite personal and professional qualifications for the new work are present, it is clear that the functions of the school medical officer may be exercised by a medical officer of health, a Poor-law medical officer, a private practitioner, or, as occasion requires, a skilled specialist. When it is necessary to appoint officers for the purpose of the Act it is extremely important that persons of suitable qualifications and experience should be selected, even though they may not be called upon to give the whole of their time to these duties, and it should be noted that there are many cases in which women are likely to be specially suitable. In making such appointments preference should be given to medical men and women who (1) have had adequate training in State medicine or hold a diploma in public health, (2) have had some definite experience of school hygiene, and (3) have enjoyed special opportunities for the study of diseases in children.

It is unnecessary to revive a controversy as to the wisdom of selecting existing Public Health organisation as the appropriate executive for the discharge of these ampler functions. We imagine that the clear statement of the Memorandum will leave only wonder that any alternative should ever have been proposed.

Not less succinct is the indication as to the scope and thoroughness of the medical inspection acceptable to the Board.

The character and degree of medical inspection will depend on the standpoint from which the subject is viewed, the difficulty being of course to attain a due sense of proportion and uniformity, particularly as to fundamental points. Valuable to science though the findings of a more thorough and elaborate medical examination might be, it is the broad, simple necessities of a healthy life which must be kept in view. It cannot be doubted that a large proportion of the common diseases and physical unfitness in this country can be substantially diminished by effective public health administration, combined with the teaching of hygiene and a realisation by teachers, parents, and children of its vital importance. The spread of communicable diseases must be checked; children's heads and bodies must be kept clean; the commoner and more obvious physical defects, at least, must be relieved, remedied, or prevented; schoolrooms must be maintained in cleanly condition, and they must be properly lighted, well ventilated, and not overcrowded; the training of the mental faculties must not be divorced from physical culture and personal hygiene. It is these primary requirements which must first receive attention.

Withal nothing of the nature of a stereotyped system is to be imposed.

The Board are desirous that the administrative machinery necessary for the appropriate working out, in various localities, of these and allied questions shall be the outcome of real organic growth rather than of a hasty attempt to impose one mechanical system upon all districts, irrespective of their requirements or resources.

Yet there is a plain warning to those Local Authorities who from any cause may seek to escape their obligations under the Act.

Conclusion and a Criticism.

The directions given in this circular as to the degree and frequency of inspection refer only to the minimum medical inspection, the effectiveness of which will in future be one of the elements to be considered in determining the efficiency of each school as a grant-aided school.

And this raises the one criticism we are prepared to offer. Medical men will appreciate at once the urgency and importance of this work, which they for long have felt was imperatively called for. Indifference and antagonism are to be met with from the Local Authorities on whom the somewhat costly burden of providing the local administration falls. To them it will mean additional expenditure out of the rates, and in many cases it will mean nothing more. The Councillors who constitute the Local Authorities for the most part bear an extraordinary resemblance to Peter Bell. The fact that there is no remission for the inevitable increase of local burdens by additional contributions from the Imperial Exchequer can hardly, however, be laid to the charge of the Board of Education. When Parliament decided upon this most necessary measure, there can be no question that it should have faced the financial aspect at the same time. An Imperial need, a national benefit, it should not have been left a mere local burden.

